# Dr. Edward Maynard

**Published:** 1891-06

**Authors:** 


THE DEATH OF DR. EDWARD MAYNARD at the ripe age of 78 years has brought to its close a long career distinguished for usefulness and crowned with abundant honor. Although he was best known to the sportsmen of America as the inventor of the arm which bears his name, Dr. Maynard's work in this field formed but a portion of his mechanical and scientific achievements. We print today an appreciative sketch of his life from the pen of Mr. H. W. S. Cleveland.—*Forest and Stream.*

